# Nitric oxide mediates interleukin-1 induced inhibition of glycosaminoglycan synthesis in rat articular cartilage

**DOI:** 10.1155/S0962935195000184

**Published:** 1995-03

**Authors:** T. A. H. Järvinen, T. Moilanen, T. L. N. Järvinen, E. Moilanen

**Affiliations:** 1 Medical School University of Tampere P.O. Box 607 Tampere SF-33101 Finland; 2 Department of Surgery University Hospital of Tampere P.O. Box 2000 Tampere SF-33521 Finland; 3 Department of Clinical Pharmacology University Hospital of Tampere P.O. Box 2000 Tampere SF-33521 Finland

## Abstract

Interleek-1β (IL-1) is a key mediator of cartilage matrix degradation in osteoarthritis and rheumatoid arthritis. It was found that the IL-1-induced suppression of glycosaminoglycan (GAG) synthesis in rat articular cartilage occurred simultaneously with the accumulation of nitrite (a metabolite of nitric oxide (NO) in aqueous milieu) in the culture medium. NO-synthase inhibitors, L-NMMA and L-NIO, inhibited both these IL-1 effects. Dexamethasone suppressed GAG synthesis additively to IL-1, but did not alter nitrite accumulation. Three NO-donors (GEA 3175, SNAP and SIN-1) also had an inhibitory effect on cartilage GAG synthesis. Therefore, it is concluded that IL-1 induced suppression of GAG synthesis in rat articular cartilage is mediated by the production of NO.


Mediators of Inflammation 4, 107–111 (1995)

### Nitric oxide mediates interleukin-1 induced inhibition of glycosaminoglycan synthesis in rat articular cartilage

T. A. H. Järvinen,[1] T. Moilanen,[2] T. L. N. Järvinen[1] and E. Moilanen[1,3,CA]

[1]Medical School, University of Tampere, P.O. Box 607, SF-33101 Tampere, Finland; [2]Departments of Surgery and [3]Clinical Pharmacology, University Hospital of Tampere, P.O. Box 2000, SF-33521 Tampere, Finland

[CA] Corresponding Author

**Interleukin-1β (IL-1) is a key mediator of cartilage matrix degradation in osteoarthritis and rheumatoid arthritis. It was found that the IL-1-induced suppression of glycosaminoglycan (GAG) synthesis in rat articular cartilage occurred simultaneously with the accumulation of nitrite (a metabolite of nitric oxide (NO) in aqueous milieu) in the culture medium. NO-synthase inhibitors, L-NMMA and L-NIO, inhibited both these IL-1 effects. Dexamethasone suppressed GAG synthesis additively to IL-1, but did not alter nitrite accumulation. Three NO-donors (GEA 3175, SNAP and SIN-1) also had an inhibitory effect on cartilage GAG synthesis. Therefore, it is concluded that IL-1 induced suppression of GAG synthesis in rat articular cartilage is mediated by the production of NO.**

**Key words**: Arthritis, Cartilage, Chondrocyte, Glucocorticoid, Glycosaminoglycan synthesis, Interleukin-1β, Nitric oxide

### Introduction

Loss of matrix proteoglycans from articular cartilage is one of the key events in the early stages of destructive joint diseases such as osteoarthritis (OA) and rheumatoid arthritis (RA). This leads to decreased resistance of articular cartilage against compressive load, which is followed by development of fissures and progressive destruction of articular cartilage. Interleukin-1 (IL-1) is a cytokine known to decrease proteoglycan synthesis in articular cartilage.[1–3] IL-1 also stimulates the synthesis of major cartilage degrading enzymes, collagenase and stromelysin, in chondrocytes and synovial cells.[3–5]

Recent reports show that nitric oxide (NO) synthesis is increased in arthritic joints.[6–10] NO is synthesized from the amino acid L-arginine in a reaction catalysed by two distinct enzymes; constitutive NO synthase (cNOS) releases small amounts of NO in response to physiological stimuli while inducible NO synthase (iNOS) produces higher amounts of NO when expressed by certain cytokines or bacterial products [for reviews, see References 11–14]. IL-1 induces the synthesis of NO in several cell types including chondrocytes of articular cartilage.[15–19] Recent reports have identified NO as a mediator of certain cytotoxic effects of IL-1 on pancreatic β cells and ovary.[20,21] The actions of NO in articular cartilage destruction are not known, but the anti-proliferative effects on chondrocytes in a co-culture with activated lymphocytes are attributed to the production of NO.[18]

Of interest to rheumatologists is the growing evidence implicating NO in inflammation, immune regulation and autoimmunity.[22,23] However, the pathophysiological role of this locally synthesized NO in OA and RA is not known. The present study was designed to test the hypothesis that NO is involved in the IL-1 induced inhibition of glycosaminoglycan (GAG) synthesis in articular cartilage. The present results show that NO acts as a mediator of the IL-1 induced inhibition of GAG synthesis in rat articular cartilage. A preliminary account of this work has been published previously.[24]

### Materials and Methods

*Animals:* Twelve-week-old male Wistar rats were used.

*Reagents and medium:* IL-1β (Immugenex, Los Angeles, USA), 3-morpholino-sydnonimine (SIN-1), *S*-nitroso-*N*-acetyl-penicillamine (SNAP) and a novel 3-aryl-substituted oxatriazol derivate GEA 3175[25–27] (GEA Ltd, Copenhagen, Denmark), *N*-monomethyl-L-arginine (L-NMMA) (Clinalfa, Läufelfingen, Switzerland), L-*N*-iminoethyl-ornithine (L-NIO) (Alexis Corp., Läufelfingen, Switzerland), dexamethasone, (Orion Pharmaceuticals, Helsinki, Finland), [$^{35}$S]-sodium sulphate (Du Pont, NEN Research Products, Boston, MA, USA), Hanks' balanced salt solution (GIBCO,

© 1995 Rapid Communications of Oxford Ltd

Grand Island, NY, USA), L-arginine (Sigma, St Louis, USA) and Lumasolve (Lumac, Basel, Switzerland) were used. RPMI 1640 (GIBCO, Grand Island, NY, USA) containing 10% heat-inactivated (56°C, 30 min) foetal bovine serum, antibiotics (100 U/ml penicillin, 100 μg/ml streptomycin and 250 ng/ml Fungizone) was used as the culture medium.

*Articular cartilage:* Culture of rat femoral head cartilage was performed according to the modified method of van den Berg *et al.*[28] Briefly, the femoral heads were removed aseptically and incubated at 37°C in a humidified 5% carbon dioxide atmosphere for 24 h. At the beginning of incubation IL-1 and/or drugs were added. After 12 h incubation equivalent concentrations of NO-donors or NO synthase inhibitors were added.

*GAG synthesis:* GAG synthesis in articular cartilage was measured by the incorporation of [$^{35}$S]-sulphate into the cartilage as described.[28] [$^{35}$S]-sodium sulphate (1 μM) was added to the culture medium and after 24 h incubation, the femoral heads were washed, fixed in ethanol and decalcified in 5% formic acid. After decalcification bone was punched out and articular cartilage digested in Lumasolve at 60°C. The amount of incorporated radioactivity was assayed by using a liquid scintillation counter. After the bony part of the femoral head had been removed, the samples were weighed. The average weight of samples was 17.13 ± 0.19 mg (mean ± S.E.M.) ($n$ = 60).

*Nitrite assay:* The incubation medium was collected after the incubation period of 24 h. Nitrite ($NO_2^-$), a stable product of NO in aqueous solutions,[29] was used as an indicator of nitric oxide.

### Results

IL-1 (0.3–30 ng/ml) decreased the cartilage GAG synthesis in a dose-dependent manner (Fig. 1). Simultaneous accumulation of nitrite in the culture medium was observed and the linear correlation coefficient between GAG synthesis and nitrite concentration was −0.63 ($p$ < 0.001, $n$ = 24). Non-stimulated cartilage samples did not release measurable concentrations of nitrite.

To assure the association of NO synthesis with the inhibitory effects of IL-1 on GAG synthesis, the effects of two competitive inhibitors of NO synthesis were studied. At concentrations needed to achieve a major inhibition of nitrite accumulation, L-NMMA (1000 μM) and L-NIO (50 μM) themselves caused a 16% and 17% reduction in GAG synthesis. L-NMMA and L-NIO diminished both IL-1 (3 ng/ml)-induced nitrite accumulation and inhibition of GAG synthesis (Fig. 2). IL-1 alone caused 33% inhibition of GAG synthesis. In the presence of L-NMMA (1000 μM) the reduction due to IL-1 was only 2%. The corresponding inhibitory effect of IL-1 in the presence of L-NIO (50 μM) was 6%. When L-NMMA or L-NIO were added with IL-1 into the organ culture, the nitrite levels in the medium were <1 μM and 1.7 μM after 24 h incubation, respectively.

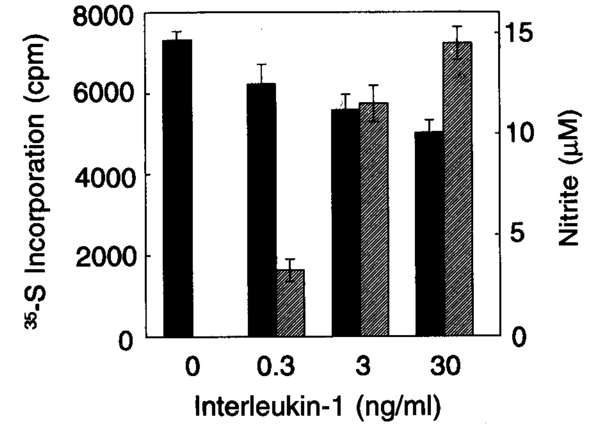

FIG. 1. The dose-dependent effects of IL-1 on articular cartilage GAG synthesis (black bars) and nitrite accumulation in the culture medium (shaded bars). Articular cartilage samples were incubated for 24 h in the presence of IL-1 (0.3–30 ng/ml). GAG and $NO_2^-$ synthesis were determined by [$^{35}$S] sulphate incorporation and Griess reaction, respectively. Results are expressed as the mean ± S.E.M. ($n$ = 6).

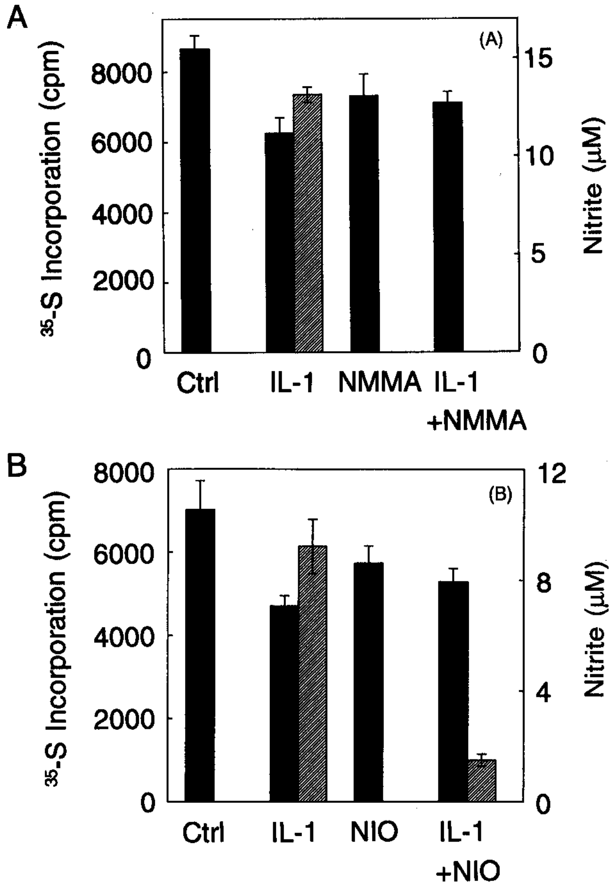

FIG. 2. The effects of IL-1 (3 ng/ml) on articular cartilage GAG synthesis (black bars) and nitrite accumulation (shaded bars) in the presence or absence of two competitive inhibitors of NO synthesis. Panel A, L-NMMA (1000 μM). Panel B, L-NIO (50 μM). GAG and $NO_2^-$ synthesis were determined by [$^{35}$S] sulphate incorporation and by Griess reaction after an incubation period of 24 h. Results are expressed as the mean ± S.E.M. ($n$ = 6).

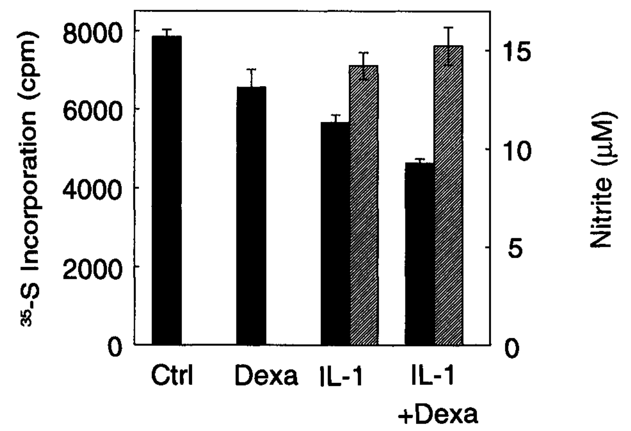

FIG. 3. The effect of IL-1 (3 ng/ml) on articular cartilage GAG synthesis (black bars) and nitrite accumulation (shaded bars) in the culture medium in the presence or absence of dexamethasone (Dexa, 1.0 µM). GAG and $NO_2^-$ synthesis were determined by [$^{35}$S] sulphate incorporation and Griess reaction after an incubation period of 24 h. Results are expressed as the mean ± S.E.M. ($n$ = 6).

Addition of L-arginine (30–1000 µM), the substrate of NO synthesis, could not induce NO synthesis alone, but suppressed nitrite accumulation dose-dependently in the presence of IL-1 (1 ng/ml) into the culture medium. At high concentrations, L-arginine (>30 µM) alone inhibited GAG synthesis as did L-NMMA and L-NIO (data not shown).

Dexamethasone was an inhibitor of GAG biosynthesis, as evidenced by 1.0 µM drug concentration which had a negative impact of 16% on GAG synthesis. When combined with IL-1 (3 ng/ml), dexamethasone had an additive inhibitory effect on GAG synthesis (Fig. 3). Dexamethasone did not alter IL-1 induced production of NO in intact articular cartilage. Neither could it alone induce any measurable nitrite accumulation. These findings were confirmed by a similar procedure using ten-fold higher concentrations of dexamethasone (10 µM) (data not shown).

The effects of three NO-releasing compounds, SIN-1, SNAP and a novel oxatriazole derivative GEA 3175, on GAG synthesis were studied. A concentration-dependent inhibition was caused by each of the three compounds in the order of potency GEA 3175 >SNAP > SIN-1. The inhibitory effect plateaued at 24–33% inhibition (Fig. 4).

### Discussion

IL-1 is a well-established mediator of inflammation and cartilage destruction in both OA and RA.[5,31] Within the cartilage IL-1 is known to promote cartilage destruction by inducing the synthesis of the major cartilage matrix degrading proteases, stromelysin and collagenase.[3–5] In addition, the inhibitory effect of IL-1 on the synthesis of new GAGs is well documented[1–3,32,33] and the treatment of experimental arthritis with anti-IL-1 antibodies could significantly suppress inflammation, decrease the enhanced cartilage breakdown and normalize chondrocyte synthetic function.[33–36] Our findings concerning IL-1 induced inhibition of the GAG biosynthesis are well in keeping with those of earlier studies,[32] even though the most recent studies[2,37] measure almost twice as large an inhibition of GAG synthesis as was found in this study. This difference is probably due to methodological differences between the studies. In the present work the radiolabelled sulphate was added to the culture concomitantly with IL-1 whereas in most of the recent studies it was added after the 6–12 h lag period needed to achieve the inhibitory action of IL-1 on GAG synthesis.[2,37]

In its target cells IL-1 binds to specific cell surface receptors and the expression and suppression of a wide range of genes takes place.[38] Even though the biological effects of IL-1 on GAG synthesis in articular cartilage âre well known, the second messenger cascade triggered by IL-1 remains obscure.

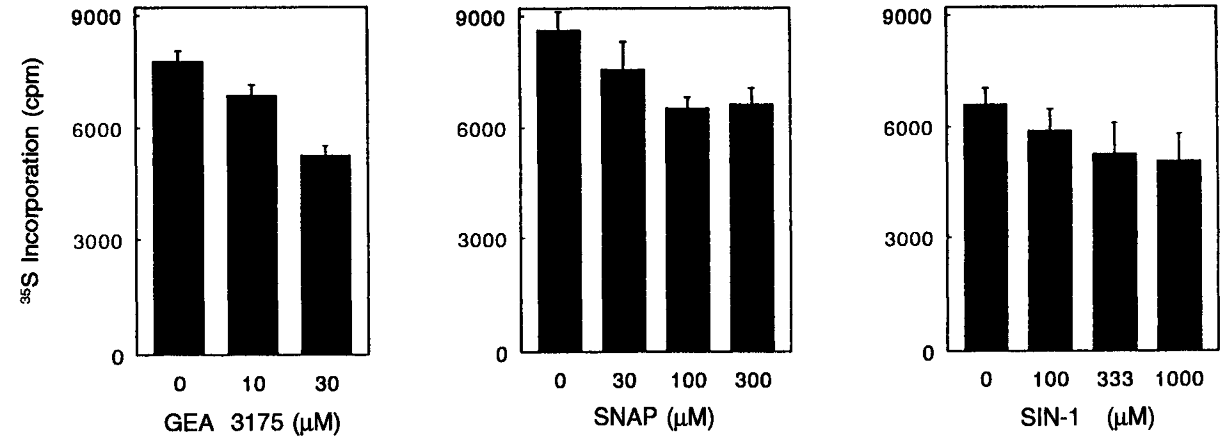

FIG. 4. The dose-dependent inhibitory effect of exogenous NO in the form of NO-releasing compounds GEA 3175, SNAP and SIN-1 on GAG synthesis in rat articular cartilage. GAG synthesis was determined by [$^{35}$S] sulphate incorporation after an incubation period of 24 h. Results are expressed as the mean ± S.E.M. ($n$ = 6–10).

Induction of iNOS by IL-1 has been shown to take place in certain cell types, including human articular chondrocytes.[15,16,19] Increased concentrations of the NO catabolite nitrite have been found in synovial fluid and sera of patients with OA or RA.[6] Four groups have recently demonstrated that increased production of NO is associated with experimental arthritis, and the administration of inhibitors of NOS suppress the reaction.[7–10] The cellular actions of NO on articular cartilage are not known. Recently, Kondo *et al.* showed that NO has an antiproliferative effect on isolated chondrocytes in a co-culture with activated lymphocytes.[18]

The production of NO by iNOS leads to inhibition of several important steps of the cellular metabolism, specifically aerobic energy metabolism, DNA replication and protein synthesis in other cell types, such as macrophages, hepatocytes, lymphocytes and tumour cells.[39–43] Recently, NO has been identified as the mediator of IL-1 induced cytotoxic effects towards pancreatic β cells and ovary cells.[20,21] The present results give several arguments to support the hypothesis that the inhibition of GAG biosynthesis in response to IL-1 is mediated by the generation of NO in the articular cartilage. First, in IL-1 stimulated articular cartilage we observed a strong association between NO production and inhibition of GAG synthesis. Secondly, when exogenous NO in the form of NO-releasing compounds (SIN-1, SNAP and GEA 3175) was added to the culture, an inhibition of GAG synthesis was seen. The two compounds with mesoinic structures, i.e. the sydnonimine SIN-1 and the recently developed oxatriazole-imine derivative GEA 3175[25–27] as well as the nitrosothiol SNAP release NO in aqueous solutions without a need of cofactors or enzymatic conversion.[44] SIN-1 releases superoxide anion ($O_2^-$) in addition to NO. These radicals can react to form peroxynitrite ($ONOO^-$) which may explain the differences in the potency of SIN-1 and compounds that release NO only.[44,45] The order of potency of these three NO-donors in the present study was similar to that shown in leukocytes and platelets.[26,27] Thirdly, the two competitive inhibitors of NO synthase, L-NMMA and L-NIO blocked IL-1 induced NO production, simultaneously preventing the IL-1 induced inhibition of GAG synthesis. The present findings accord well with those reported by Taskiran *et al.*[37] during the processing of this manuscript.

The NO production in rat articular cartilage in response to IL-1 was decreased after supplementation of medium with higher concentrations of L-arginine. This phenomenon has earlier been recorded in lapine articular chondrocytes, suggesting a rather high affinity of the chondrocyte NOS for the endogenous L-arginine.[15] In our study L-arginine alone, as well as L-NMMA and L-NIO, suppressed GAG synthesis thus giving additional data to the earlier suggestions of undefined toxic effects of higher concentrations of L-arginine on the articular chondrocytes[15] and L-arginine analogue inhibitors of NOS on other tissues.[12]

A number of isolforms of iNOS have been described.[14] The expression of the iNOS is inhibited by glucocorticoids in several cell types, such as macrophages and endothelial cells.[11–14] In articular cartilage, IL-1 induced production of NO was not altered by dexamethasone, confirming similar observations in human and rabbit chondrocytes.[16,17] Besides, dexamethasone had an inhibitory effect on GAG synthesis, which was additive to that of IL-1.

The clinical and subjective improvement of the RA and OA patients after glucocorticoid treatment is thought to be mediated by the inhibitory action of glucocorticoids on the synthesis of proinflammatory cytokines, prostaglandins and proteolytic enzymes.[46–49] The present findings of the additive suppressive effects of dexamethasone and IL-1 on GAG synthesis and the failure of dexamethasone to prevent the induction of NO synthesis in articular cartilage could be regarded as unfavourable events in the pathogenesis of cartilage destruction. Furthermore, these findings support the assumption of Stefanovic-Racic *et al.*[23] that the insensitivity of iNOS to glucocorticoids in articular cartilage might partly explain why anti-inflammatory steroids fail to retard the cartilage destruction in arthritic joints even though they suppress the inflammatory action.

In conclusion, these observations suggest that the effects of IL-1 and glucocorticoids on rat articular cartilage GAG biosynthesis are mediated by different intracellular mechanisms, which in the case of IL-1 seems to be NO.

### References

1. Tyler JA. Articular cartilage cultured with catabolin (pig interleukin 1) synthesizes a decreased number of normal proteoglycan molecules. *Biochem J* 1985; **227**: 869–878.
2. Benton HP, Tyler JA. Inhibition of cartilage proteoglycan synthesis by interleukin 1. *Biochem Biophys Res Commun* 1988; **154**: 421–428.
3. van den Berg WB, van de Loo AAJ, Zwarts WA, Otterness IG. Effects of murine recombinant IL-1 on intact homologous articular cartilage: a quantitative and autoradiographic study. *Ann Rheum Dis* 1988; **47**: 855–863.
4. Murphy G, Hembry RM, Reynolds JJ. Characterization of a specific antiserum to rabbit stromelysin and demonstration of the synthesis of collagenase and stromelysin by stimulated rabbit articular chondrocytes. *Coll Relat Res* 1986; **6**: 351–364.
5. Arend WP, Dayer JM. Cytokines and cytokine inhibitors or antagonists in rheumatoid arthritis. *Arthritis Rheum* 1990; **33**: 305–315.
6. Farrell AJ, Blake DR, Palmer RMJ, Moncada S. Increased concentrations of nitrite in synovial fluid and serum samples suggest increased nitric oxide synthesis in rheumatic diseases. *Ann Rheum Dis* 1992; **51**: 1219–1222.
7. McCartney-Francis N, Allen JB, Mizel DE, *et al.* Suppression of arthritis by an inhibitor of nitric oxide synthase. *J Exp Med* 1993; **178**: 749–754.
8. Ialenti A, Moncada S, Di Rosa M. Modulation of adjuvant arthritis by endogenous nitric oxide. *Br J Pharmacol* 1993; **110**: 701–706.
9. Weinberg JB, Granger DL, Pisetsky DS, *et al.* The role of nitric oxide in the pathogenesis of spontaneous murine autoimmune disease: increased nitric oxide synthase expression in MRL-lpr/lpr mice, and reduction of spontaneous glomerulonephritis and arthritis by orally administered $N^G$-monomethyl-L-arginine. *J Exp Med* 1994; **179**: 651–660.
10. Stefanovic-Racic M, Meyers K, Meschter C, Coffey JW, Hoffman RA, Evans CH. *N*-monomethyl arginine, an inhibitor of nitric oxide synthase, suppresses the development of adjuvant arthritis in rats. *Arthritis Rheum* 1994; **37**: 1062–1069.

11. Moncada S. The 1991 Ulf von Euler lecture. The L-arginine: nitric oxide pathway. *Acta Physiol Scand* 1992; **145**: 201–227.
12. Nathan C. Nitric oxide as a secretory product of mammalian cells. *FASEB J* 1992; **6**: 3051–3064.
13. Moncada S, Higgs EA. The L-arginine–nitric oxide pathway. *N Engl J Med* 1993; **329**: 2002–2012.
14. Knowles RG, Moncada S. Nitric oxide synthases in mammals. *Biochem J* 1994; **298**: 249–258.
15. Stadler J, Stefanovic-Racic M, Billiar TR, *et al.* Articular chondrocytes synthesize nitric oxide in response to cytokines and lipopolysaccharide. *J Immunol* 1991; **147**: 3915–3920.
16. Palmer RMJ, Andrews T, Foxwell NA, Moncada S. Glucocorticoids do not affect the induction of a novel calcium-dependent nitric oxide synthase in rabbit chondrocytes. *Biochem Biophys Res Comm* 1992; **188**: 209–215.
17. Palmer RMJ, Hickery MS, Charles IG, Moncada S, Bayliss MT. Induction of nitric oxide synthase in human chondrocytes. *Biochem Biophys Res Comm* 1993; **193**: 398–405.
18. Kondo S, Ishiguro N, Iwata H, Nakashima I, Isobe K. The effects of nitric oxide on chondrocytes and lymphocytes. *Biochem Biophys Res Comm* 1993; **197**: 1431–1437.
19. Charles IG, Palmer RMJ, Hickery MS, *et al.* Cloning, characterization, and expression of a cDNA encoding an inducible nitric oxide synthase from the human chondrocyte. *Proc Natl Acad Sci USA* 1993; **90**: 11419–11423.
20. Corbett JA, McDaniel M. Does nitric oxide mediate autoimmune destruction of β cells? *Diabetes* 1992; **41**: 897–903.
21. Ellman C, Corbett JA, Misko TP, McDaniel M, Beckerman KP. Nitric oxide mediates interleukin-1-induced cellular cytotoxicity in the rat ovary. A potential role for nitric oxide in the ovulatory process. *J Clin Invest* 1993; **86**: 3053–3065.
22. Nussler AK, Billiar TR. Inflammation, immunoregulation, and inducible nitric oxide synthase. *J Leukoc Biol* 1993; **54**: 171–178.
23. Stefanovic-Racid M, Stadler J, Evans CH. Nitric oxide and arthritis. *Arthritis Rheum* 1993; **36**: 1036–1044.
24. Järvinen TAH, Moilanen T, Järvinen TLN, Moilanen E. Nitric oxide mediates interleukin-1 induced inhibition of glycosaminoglycan synthesis in rat articular cartilage. *Scand J Rheumatol* 1994; **25**: 163a.
25. Robak J, Macrinkiewicz E, Gryglewski R, Correll TM, Petersen SB, Karup G. Scavenging of superoxide anions by nitric oxide donors. *Pharmacol Res* 1992; **25** (suppl 2): 355–356.
26. Moilanen E, Arola O, Malo-Ranta U, *et al.* Nitric oxide donors inhibit neutrophil adhesion to endothelial cells. In: Moncada S, Feelish M, Busse R, Higgs EA, eds. *The Biology of Nitric Oxide, Vol 4.* 1994; 271–275.
27. Corell T, Petersen SB, Lissau B, *et al.* Pharmacology of mesoionic oxatriazole derivatives in blood, cardiovascular and respiratory systems. *Pol J Pharmacol* (in press).
28. van den Berg WB, Kruijsen MWM, van de Putte LBA. The mouse patella assay. An easy method of quantitating articular cartilage chondrocyte function *in vivo* and *in vitro*. *Rheumatol Int* 1982; **1**: 165–169.
29. Ignarro LJ, Fukuto JM, Griscavage JM, Rogers NE, Byrns RE. Oxidation of nitric oxide in aqueous solution to nitrite but not nitrate: comparison with enzymatically formed nitric oxide from L-arginine. *Proc Natl Acad Sci USA* 1993; **90**: 8103–8107.
30. Green LC, Wagner DA, Glowgowski J, Skepper PL, Wishnok JS, Tannenbaum SR. Analysis of nitrate, nitrite and [15N] nitrate in biological fluids. *Anal Biochem* 1982; **124**: 131–138.
31. Cameron ML, Fu FH, Paessler HH, Schneider M, Evans CH. Synovial fluid cytokine concentrations as possible prognostic indicators in the ACL-deficient knee. *Knee Surg, Sports Traumatol, Arthroscopy* 1994; **2**: 38–44.
32. Pratta MA, Di Meo TM, Rihl DM, Arner EC. Effect of interleukin-1-β and tumor necrosis factor-α on cartilage proteoglycan metabolism *in vitro*. *Agents Actions* 1989; **27**: 249–253.
33. van de Loo FAJ, Arntz OJ, Otterness IG, van de Berg WB. Protection against cartilage proteoglycan synthesis inhibition by antiinterleukin 1 antibodies in experimental arthritis. *J Rheumatol* 1992; **19**: 348–356.
34. van den Berg WB, Joosten LAB, Helsen M, van de Loo FAJ. Amelioration of established murine collagen-induced arthritis with anti-IL-1 treatment. *Clin Exp Immunol* 1994; **95**: 237–243.
35. van de Loo FAJ, Joosten LAB, van Lent PLEM, Arntz OJ, van den Berg WB. Role of interleukin-1 ((IL-1), tumor necrosis factor (TNF)-α and interleukin-6 (IL-6) in cartilage proteoglycan metabolism and destruction: effect of *in situ* blocking in murine antigen- and zymosan induced arthritis. *Arthritis Rheum* 1995; **38**: 164–172.
36. van de Loo AAJ, Arntz OJ, Bakker AC, van Lent PLEM, Jacobs MJM, van den Berg WB. Role of interleukin-1 (IL-1) in antigen-induced exacerbations of murine arthritis. *Am J Pathol* 1995; **38**: 164–172..
37. Taskiran D, Stefanovic-Racid M, Georgescu H, Evans C. Nitric oxide mediates suppression of cartilage proteoglycan synthesis by interleukin-1 *Biochem Biophys Res Comm* 1994; **200**: 142–148.
38. Dinarello CA, Wolff SM. Role of interleukin-1 in disease. *N Engl J Med* 1993; **328**: 106–113.
39. Stuehr DJ, Nathan CF. Nitric oxide: a macrophage product responsible for cytostasis and respiratory inhibition in tumor target cells. *J Exp Med* 1989; **169**: 1543–1555.
40. Stadler J, Billiar TR, Curran RD, Stuehr DJ, Ochoa JB, Simmons RL. Effect of exogenous and endogenous nitric oxide on mitochondrial respiration of rat hepatocytes. *Am J Physiol* 1991; **260**: C910–C916.
41. Hoffman RA, Langrehn JM, Billiar TR, Curran RD, Simmons RL. Alloantigen-induced activation of rat splenocytes is regulated by the oxidative metabolism of L-arginine. *J Immunol* 1991; **146**: 2719–2723.
42. Lepoivre M, Chenais B, Yapo A, Lemaire G, Thelander L, Tenu JP. Alteration of ribonucleotide reductase activity following induction of the nitrite-generating pathway in adenocarcinoma cells. *J Biol Chem* 1990; **265**: 14143–14149.
43. Curan RD, Ferrari FK, Kispert PH, *et al.* Nitric oxide and nitric oxide-generating compounds inhibit hepatocyte protein synthesis. *FASEB J* 1991; **5**: 2085–2092.
44. Feelish M. The biochemical pathways of nitric oxide formation from nitrovasodilators: appropriate choice of exogenous NO donors and aspects of preparation and handling of aqueous solutions. *J Cardiovasc Pharmacol* 1991; **17**(suppl 3): S25–S33.
45. Hogg N, Darley-Usmar VM, Wilson MT, Moncada S. Production of hydroxyl radicals from simultaneous generation of superoxide and nitric oxide. *Biochem J* 1992; **281**: 419–424.
46. Kirkham B. Interleukin-1, immune activation pathways, and different mechanisms in osteoarthritis and rheumatoid arthritis. *Ann Rheum Dis* 1991; **50**: 395–400.
47. Crofford LJ, Wilder RL, Ristimäki AP, *et al.* Cycloxygenase-1 and -2 expression in rheumatoid synovial tissues. Effects of interleukin-1β, phorbol ester, and corticosteroids. *J Clin Invest* 1994; **93**: 1095–1101.
48. Hla T, Ristimäki A, Appleby S, Barriocanal JG. Cyclooxygenase gene expression in inflammation and angiogenesis. *Ann NY Acad Sci* 1993; **696**: 197–204.
49. Martel-Pelletier J, McCollum R, Fujimoto N, Obata K, Cloutier J-M, Pelletier J-M. Excess of metalloprotease over tissue inhibitor of metalloprotease may contribute to cartilage degradation in osteoarthritis and rheumatoid arthritis. *Lab Invest* 1994; **70**: 807–815.

**Received 28 September 1994; accepted in revised form 1 December 1994**

## References

[PDF_00397] Arend W. P., Dayer J. M. (1990). Cytokines and cytokine inhibitors or antagonists in rheumatoid arthritis.. Arthritis Rheum.

[PDF_00388] Benton H. P., Tyler J. A. (1988). Inhibition of cartilage proteoglycan synthesis by interleukin I.. Biochem Biophys Res Commun.

[PDF_00468] Cameron M. L., Fu F. H., Paessler H. H., Schneider M., Evans C. H. (1994). Synovial fluid cytokine concentrations as possible prognostic indicators in the ACL-deficient knee.. Knee Surg Sports Traumatol Arthrosc.

[PDF_00435] Charles I. G., Palmer R. M., Hickery M. S., Bayliss M. T., Chubb A. P., Hall V. S., Moss D. W., Moncada S. (1993). Cloning, characterization, and expression of a cDNA encoding an inducible nitric oxide synthase from the human chondrocyte.. Proc Natl Acad Sci U S A.

[PDF_00438] Corbett J. A., McDaniel M. L. (1992). Does nitric oxide mediate autoimmune destruction of beta-cells? Possible therapeutic interventions in IDDM.. Diabetes.

[PDF_00514] Crofford L. J., Wilder R. L., Ristimäki A. P., Sano H., Remmers E. F., Epps H. R., Hla T. (1994). Cyclooxygenase-1 and -2 expression in rheumatoid synovial tissues. Effects of interleukin-1 beta, phorbol ester, and corticosteroids.. J Clin Invest.

[PDF_00503] Curran R. D., Ferrari F. K., Kispert P. H., Stadler J., Stuehr D. J., Simmons R. L., Billiar T. R. (1991). Nitric oxide and nitric oxide-generating compounds inhibit hepatocyte protein synthesis.. FASEB J.

[PDF_00490] Dinarello C. A., Wolff S. M. (1993). The role of interleukin-1 in disease.. N Engl J Med.

[PDF_00440] Ellman C., Corbett J. A., Misko T. P., McDaniel M., Beckerman K. P. (1993). Nitric oxide mediates interleukin-1-induced cellular cytotoxicity in the rat ovary. A potential role for nitric oxide in the ovulatory process.. J Clin Invest.

[PDF_00399] Farrell A. J., Blake D. R., Palmer R. M., Moncada S. (1992). Increased concentrations of nitrite in synovial fluid and serum samples suggest increased nitric oxide synthesis in rheumatic diseases.. Ann Rheum Dis.

[PDF_00517] Hla T., Ristimäki A., Appleby S., Barriocanal J. G. (1993). Cyclooxygenase gene expression in inflammation and angiogenesis.. Ann N Y Acad Sci.

[PDF_00509] Hogg N., Darley-Usmar V. M., Wilson M. T., Moncada S. (1992). Production of hydroxyl radicals from the simultaneous generation of superoxide and nitric oxide.. Biochem J.

[PDF_00404] Ialenti A., Moncada S., Di Rosa M. (1993). Modulation of adjuvant arthritis by endogenous nitric oxide.. Br J Pharmacol.

[PDF_00462] Ignarro L. J., Fukuto J. M., Griscavage J. M., Rogers N. E., Byrns R. E. (1993). Oxidation of nitric oxide in aqueous solution to nitrite but not nitrate: comparison with enzymatically formed nitric oxide from L-arginine.. Proc Natl Acad Sci U S A.

[PDF_00512] Kirkham B. (1991). Interleukin-1, immune activation pathways, and different mechanisms in osteoarthritis and rheumatoid arthritis.. Ann Rheum Dis.

[PDF_00422] Knowles R. G., Moncada S. (1994). Nitric oxide synthases in mammals.. Biochem J.

[PDF_00433] Kondo S., Ishiguro N., Iwata H., Nakashima I., Isobe K. (1993). The effects of nitric oxide on chondrocytes and lymphocytes.. Biochem Biophys Res Commun.

[PDF_00500] Lepoivre M., Chenais B., Yapo A., Lemaire G., Thelander L., Tenu J. P. (1990). Alterations of ribonucleotide reductase activity following induction of the nitrite-generating pathway in adenocarcinoma cells.. J Biol Chem.

[PDF_00519] Martel-Pelletier J., McCollum R., Fujimoto N., Obata K., Cloutier J. M., Pelletier J. P. (1994). Excess of metalloproteases over tissue inhibitor of metalloprotease may contribute to cartilage degradation in osteoarthritis and rheumatoid arthritis.. Lab Invest.

[PDF_00402] McCartney-Francis N., Allen J. B., Mizel D. E., Albina J. E., Xie Q. W., Nathan C. F., Wahl S. M. (1993). Suppression of arthritis by an inhibitor of nitric oxide synthase.. J Exp Med.

[PDF_00420] Moncada S., Higgs A. (1993). The L-arginine-nitric oxide pathway.. N Engl J Med.

[PDF_00416] Moncada S. (1992). The 1991 Ulf von Euler Lecture. The L-arginine: nitric oxide pathway.. Acta Physiol Scand.

[PDF_00393] Murphy G., Hembry R. M., Reynolds J. J. (1986). Characterization of a specific antiserum to rabbit stromelysin and demonstration of the synthesis of collagenase and stromelysin by stimulated rabbit articular chondrocytes.. Coll Relat Res.

[PDF_00418] Nathan C. (1992). Nitric oxide as a secretory product of mammalian cells.. FASEB J.

[PDF_00443] Nussler A. K., Billiar T. R. (1993). Inflammation, immunoregulation, and inducible nitric oxide synthase.. J Leukoc Biol.

[PDF_00430] Palmer R. M., Hickery M. S., Charles I. G., Moncada S., Bayliss M. T. (1993). Induction of nitric oxide synthase in human chondrocytes.. Biochem Biophys Res Commun.

[PDF_00471] Pratta M. A., Di Meo T. M., Ruhl D. M., Arner E. C. (1989). Effect of interleukin-1-beta and tumor necrosis factor-alpha on cartilage proteoglycan metabolism in vitro.. Agents Actions.

[PDF_00494] Stadler J., Billiar T. R., Curran R. D., Stuehr D. J., Ochoa J. B., Simmons R. L. (1991). Effect of exogenous and endogenous nitric oxide on mitochondrial respiration of rat hepatocytes.. Am J Physiol.

[PDF_00411] Stefanovic-Racic M., Meyers K., Meschter C., Coffey J. W., Hoffman R. A., Evans C. H. (1994). N-monomethyl arginine, an inhibitor of nitric oxide synthase, suppresses the development of adjuvant arthritis in rats.. Arthritis Rheum.

[PDF_00445] Stefanovic-Racic M., Stadler J., Evans C. H. (1993). Nitric oxide and arthritis.. Arthritis Rheum.

[PDF_00492] Stuehr D. J., Nathan C. F. (1989). Nitric oxide. A macrophage product responsible for cytostasis and respiratory inhibition in tumor target cells.. J Exp Med.

[PDF_00487] Taskiran D., Stefanovic-Racic M., Georgescu H., Evans C. (1994). Nitric oxide mediates suppression of cartilage proteoglycan synthesis by interleukin-1.. Biochem Biophys Res Commun.

[PDF_00385] Tyler J. A. (1985). Articular cartilage cultured with catabolin (pig interleukin 1) synthesizes a decreased number of normal proteoglycan molecules.. Biochem J.

[PDF_00406] Weinberg J. B., Granger D. L., Pisetsky D. S., Seldin M. F., Misukonis M. A., Mason S. N., Pippen A. M., Ruiz P., Wood E. R., Gilkeson G. S. (1994). The role of nitric oxide in the pathogenesis of spontaneous murine autoimmune disease: increased nitric oxide production and nitric oxide synthase expression in MRL-lpr/lpr mice, and reduction of spontaneous glomerulonephritis and arthritis by orally administered NG-monomethyl-L-arginine.. J Exp Med.

[PDF_00474] van de Loo F. A., Arntz O. J., Otterness I. G., van den Berg W. B. (1992). Protection against cartilage proteoglycan synthesis inhibition by antiinterleukin 1 antibodies in experimental arthritis.. J Rheumatol.

[PDF_00480] van de Loo F. A., Joosten L. A., van Lent P. L., Arntz O. J., van den Berg W. B. (1995). Role of interleukin-1, tumor necrosis factor alpha, and interleukin-6 in cartilage proteoglycan metabolism and destruction. Effect of in situ blocking in murine antigen- and zymosan-induced arthritis.. Arthritis Rheum.

[PDF_00477] van den Berg W. B., Joosten L. A., Helsen M., van de Loo F. A. (1994). Amelioration of established murine collagen-induced arthritis with anti-IL-1 treatment.. Clin Exp Immunol.

[PDF_00390] van den Berg W. B., van de Loo F. A., Zwarts W. A., Otterness I. G. (1988). Effects of murine recombinant interleukin 1 on intact homologous articular cartilage: a quantitative and autoradiographic study.. Ann Rheum Dis.

